# Transesophageal Echocardiography-Guided Transseptal Puncture Reduces Pericardial Tamponade in Electrophysiological Procedures

**DOI:** 10.3390/diagnostics14222495

**Published:** 2024-11-08

**Authors:** Yannick Teumer, Daniel Eckart, Lyuboslav Katov, Dominik Felbel, Carlo Bothner, Wolfgang Rottbauer, Karolina Weinmann-Emhardt

**Affiliations:** Ulm University Heart Center, Ulm University, Albert-Einstein-Allee 23, 89081 Ulm, Germany; yannick.teumer@uniklinik-ulm.de (Y.T.); daniel.eckart@uni-ulm.de (D.E.); lyuboslav.katov@uniklinik-ulm.de (L.K.); dominik.felbel@uniklinik-ulm.de (D.F.); carlo.bothner@uniklinik-ulm.de (C.B.); wolfgang.rottbauer@uniklinik-ulm.de (W.R.)

**Keywords:** transseptal puncture, electrophysiology, transesophageal echocardiography, pericardial effusion, pericardial tamponade

## Abstract

**Background**: Transseptal puncture (TSP) is a critical step in electrophysiological (EP) procedures, as a misdirected TSP can result in life-threatening complications. Although TSP is predominantly performed under fluoroscopic guidance in EP procedures, transesophageal echocardiography (TEE) offers more precision and certainty in the localization of the transseptal needle at the interatrial septum. Despite the widespread use of TSP, evidence supporting the added value of TEE-guided TSP in EP procedures remains limited. This study evaluates the impact of additional TEE guidance on TSP-associated complications during EP procedures. **Methods**: This study enrolled patients who underwent left atrial or left ventricular procedures with TSP, performed either without (fluoroscopy group) or with additional TEE guidance (TEE group), at the University Heart Center Ulm, Germany. **Results**: A total of 932 patients were included: 443 in the TEE group (mean age 68.1 ± 11.8 years, 40.6% female) and 489 in the fluoroscopy group (mean age 68.8 ± 11.0 years, 38.2% female). The mean number of transseptal accesses per patient was 1.18 ± 0.38 in the TEE group and 1.14 ± 0.34 in the fluoroscopy group (*p* = 0.101). Pericardial tamponade occurred significantly less in the TEE group (0.5%) than in the fluoroscopy group (1.8%; *p* = 0.046). Logistic regression revealed a 91.8% lower risk of pericardial tamponade with TEE-guided TSP compared to fluoroscopy guidance alone. The overall TEE complication rate was low (0.9%). **Conclusions**: TEE guidance during TSP significantly reduces the risk of pericardial tamponade in EP procedures, indicating that TSP should be performed with additional sonographic guidance to increase patient safety.

## 1. Introduction

Transseptal puncture (TSP) is a mainstay of every endovascular left atrial and, in some cases, left ventricular procedure in interventional electrophysiology. Pericardial tamponades are considered the most common cause of fatal complications during an electrophysiological procedure [1,2]. Besides cardiac perforation by an ablation catheter and steam pops, the most frequent cause of pericardial tamponade is a misplaced TSP [2].

Despite the known risks, approximately half of all TSPs during electrophysiological procedures are still performed under solely fluoroscopic guidance [2,3]. However, additional intraprocedural echocardiographic imaging, such as transesophageal or intracardiac echocardiography, offers advantages over fluoroscopy alone. These techniques provide a real-time visualization of the patient’s individual anatomical structures, allowing for the more accurate localization of the transseptal needle and more precise determination of the puncture site [4,5].

In many countries, the use of intracardiac echocardiography (ICE) is limited due to additional costs and a lack of reimbursement, but transesophageal echocardiography (TEE) is often available [6,7]. While a reduction in the occurrence of pericardial effusion and tamponades has been demonstrated with ICE [8], this risk reduction has not yet been shown for TEE in direct comparison to solely fluoroscopy-guided TSP in electrophysiological procedures. Consequently, the aim of this study was to investigate the impact of additional TEE guidance on the occurrence of pericardial tamponade and effusion, in direct comparison to fluoroscopy guidance alone during TSP in electrophysiological procedures.

## 2. Materials and Methods

### 2.1. Study Design

This study included patients over 18 years of age who were enrolled in the Ulm Arrhythmia Registry and scheduled for electrophysiological procedures located in the left atrium and left ventricle via transseptal access at the University Heart Center in Germany between November 2021 and October 2023. From November 2021 to October 2022, TSP was performed using fluoroscopic guidance alone (fluoroscopy group). From November 2022 to October 2023, transseptal access was achieved with both fluoroscopic and additional TEE guidance (TEE group). Data were collected as part of the Ulm Arrhythmia Registry (German Clinical Trials Register ID: DRKS00013013). This study adheres to the Declaration of Helsinki and received approval from the local ethics committee of Ulm University (324/16).

### 2.2. Transseptal Puncture Protocol and Transesophageal Guidance

The TSP protocol at our center was described in detail before [4]. The TSP was performed solely by experienced operators. In brief, after establishing venous access in the right groin of the patient, a non-steerable transseptal sheath (CardiaGuide™, Johnson & Johnson, New Brunswick, NJ, USA) was advanced into the superior vena cava via a guidewire. The transseptal needle (HeartSpan™ Transseptal Needle™, Johnson & Johnson, New Brunswick, NJ, USA) was then inserted into the transseptal sheath without the tip of the needle protruding beyond the tip of the sheath. Following this, the assembly of the transseptal sheath and needle was withdrawn into the right atrium. The placement of the TSP system at the interatrial septum was conducted, depending on the study group, either solely fluoroscopically or with combined fluoroscopy and TEE. In fluoroscopy, the left anterior oblique (LAO) 40° view, the anteroposterior (AP) 0° view, and the right anterior oblique (RAO) 30° view were used. In TEE, a mid-esophageal position was used (Philips CX50 ultrasound system with a Philips X7 TEE probe, Philips, Amsterdam, The Netherlands), with the simultaneous visualization of orthogonal views at 45° and 135° to determine the position of the transseptal needle. The TEE was performed under conscious sedation.

### 2.3. Periprocedural Management

All procedures were conducted with uninterrupted oral anticoagulation therapy and deep sedation. Transthoracic echocardiography assessment was performed pre- and post-intervention, on the day after the procedure and before discharge.

### 2.4. Statistical Analysis

Statistical analysis and the graphical presentation of the results were carried out using SPSS Statistics (version 29.0, IBM, Armonk, NY, USA) and Excel (version 16.75, Microsoft, Redmond, DC, USA). Variables were analyzed according to their scale of measurement. Categorical variables were expressed as absolute and relative frequencies, while continuous variables were presented either as the mean ± standard deviation (SD) or the median with interquartile range, depending on the distribution of the data. For categorical variables, the Chi-square test or Fisher’s exact test was applied, while continuous variables were analyzed using the Mann–Whitney U test or Student’s *t*-test, as appropriate. A *p*-value of less than 0.05 was considered statistically significant. To evaluate the influence of various factors on the incidence of pericardial tamponade, pericardial effusion, and peri-interventional blood transfusions, binary logistic regression was conducted. The independent variables included in the regression analysis were selected based on content-related and statistical considerations. To reduce potential overfitting, parameters were reduced in the regression model using backward elimination according to the likelihood ratio method. The model’s validity was confirmed through the Omnibus test and the Hosmer–Lemeshow goodness-of-fit statistic.

### 2.5. Study Endpoints

The study endpoints related to TSP included the peri-interventional occurrence of pericardial effusion, pericardial tamponade, the need for blood transfusions after pericardiocentesis, rehospitalization due to pericardial effusion or tamponade, and fatality. Additionally, TEE-associated complications were assessed, focusing on the occurrence of peri-interventional gastrointestinal bleeding and injuries of surrounding structures caused by the TEE probe, particularly esophageal lesions, both with and without the need for treatment.

## 3. Results

A total of 932 patients were included in this study, with 443 patients in the TEE group (47.5%) and 489 patients (52.5%) in the fluoroscopy group. The sex distribution in both groups was similar (*p* = 0.613). While the partial thromboplastin time (PTT) values were within the normal range (27–35 s) for both groups, they were significantly lower in the TEE group (32.8 ± 7.3 vs. 34.6 ± 8.4 s, *p* < 0.001). In contrast, the international normalized ratio (INR) values were significantly higher in the TEE group (1.21 ± 0.36 vs. 1.18 ± 0.32, *p* = 0.032), with both groups slightly exceeding the normal range (0.85–1.15). Furthermore, no significant differences were observed between the two groups in terms of the use of platelet count, platelet aggregation inhibitors and oral anticoagulation. Detailed patient characteristics are depicted in Table 1.

### 3.1. Procedural Data

A total of 1077 transseptal accesses were established in 932 patients for this study, with a maximum of two transseptal accesses per patient. On average, there was no significant difference in the number of transseptal accesses performed during the index procedure (*p* = 0.101). Similarly, no significant difference was observed in the diameters of the transseptal sheaths used between the two study groups (*p* = 0.289). Regarding procedural parameters, the only notable difference was in the types of treated tachycardias (*p* = 0.022). In both study groups, the predominant tachycardia treated was atrial fibrillation. First-time pulmonary vein isolation (PVI) was performed in 70.5% of cases in the TEE group and 74.5% in the fluoroscopy group (*p* = 0.685). Of these, 57.1% of the PVIs in the TEE group and 59.1% in the fluoroscopy group were performed using a single-shot device (*p* = 0.535). Further procedural details are shown in Table 2.

### 3.2. Complication Data

**Figure 1:**
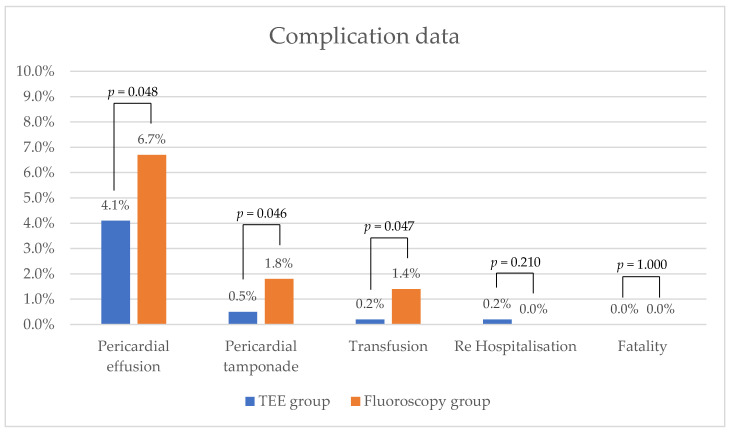
Graphical comparison of complication rates between the TEE group and the fluoroscopy group. TEE, transesophageal echocardiography.

A total of 51 pericardial effusions were detected in 932 patients. Of these, 18 (4.7%) occurred in the TEE group and 33 (6.7%) occurred in the fluoroscopy group. This indicates significantly fewer pericardial effusions per patient in the TEE group (*p* = 0.048). Similarly, there were significantly fewer pericardial tamponades requiring intervention in the TEE group (2 [0.5%] vs. 9 [1.8%], *p* = 0.046). After pericardiocentesis, blood transfusions were necessary for one patient in the TEE group and seven patients in the fluoroscopy group (*p* = 0.047). In all cases with the need for transfusion, part of the pericardial effusion was retransfused as an autotransfusion. Additionally, one patient (0.2%) in the TEE group and four patients (0.8%) in the fluoroscopy group received donor blood transfusions. Surgical intervention was not required to control bleeding in any of the patients. One patient (0.2%) in the TEE group was re-hospitalized due to a pericardial effusion, which was treated conservatively. No fatal complications occurred in either group. For further details, see Figure 1.

### 3.3. Logistic Regression

Even after adjusting the results using logistic regression with backward elimination for factors such as age, sex, body mass index, international normalized ratio, partial thromboplastin time, type of treated tachycardia, and the number of transseptal accesses per patient, a protective effect of additional TEE guidance was observed. This effect was significant in reducing the incidence of pericardial tamponade (odds ratio 0.103, confidence interval 0.013–0.819) and pericardial effusion (odds ratio 0.466, confidence interval 0.246–0.883). It was not significant regarding the need for peri-interventional blood transfusions after pericardiocentesis (odds ratio 0.132, confidence interval 0.016–1.095). The three regression models were statistically significant at *p* = 0.004, *p* < 0.001, and *p* = 0.002, respectively. For further details, see Table 3, Table 4 and Table 5 and Supplementary Tables S1–S3.

### 3.4. Complications Related to Transesophageal Echocardiography

In the TEE group, the TEE was successfully performed in 99.1% of patients. In four patients, it was not possible to guide the TSP by TEE. Two patients (0.5%) experienced bile vomiting during the introduction of the TEE probe, despite a six-hour preoperative fasting period, necessitating that the TSP be performed using fluoroscopic guidance alone. In the other two cases, the TEE probe could not be advanced into the esophagus due to anatomical reasons.

A total of four TEE-associated complications occurred in the TEE group (0.9%). Two patients (0.5%) experienced bile vomiting without aspiration at the start of the examination. One patient (0.2%) experienced a tracheal misintubation, which was promptly detected and successfully corrected by the TEE operator without further sequelae. In another patient (0.2%), hypopharyngeal bleeding occurred during the ablation procedure following the transnasal insertion of an esophageal temperature probe, necessitating protective intubation. After an esophagogastroduodenoscopy, it was not possible to distinguish between mucosal injury caused by the TEE probe or the temperature probe. Once the bleeding ceased spontaneously, the patient was successfully extubated.

## 4. Discussion

To the best of our knowledge, this is the largest study to date directly comparing the use of additional TEE guidance with fluoroscopy-only guidance for achieving transseptal access in electrophysiological procedures. Moreover, it is the first study to demonstrate a significant reduction in peri-interventional pericardial effusion and pericardial tamponade rates when TEE guidance is added, compared to fluoroscopy alone, in an electrophysiology population. This study thus answers the question regarding the influence of additional TEE during TSP on pericardial effusion and tamponade, as already requested by other authors [9].

The multivariate logistic regression revealed a clinically relevant and statistically significant reduction in the risk of pericardial effusion by 90% and pericardial tamponade by more than 50% with the additional use of TEE.

If one considers the absolute risk of pericardial effusion or tamponade of the fluoroscopy group, this is comparable with that described in the literature [2,10,11]. Interestingly, the results of this study, which demonstrate a risk reduction associated with additional echocardiographic guidance during TSP, are consistent with the risk reductions described in the literature when using ICE during electrophysiological procedures [5,8]. Therefore, it seems likely that the reduction in pericardial effusion and tamponade is related to the additional interventional imaging. Unlike fluoroscopy, TEE provides a more detailed real-time visualization of individual cardiac anatomy [12]. This allows for the better visualization of the transseptal needle at the interatrial septum during puncture, enabling a more precise selection of the puncture site [4,12,13], which may explain the reduction in pericardial tamponade rates with the additional use of TEE.

While fluoroscopy-guided TSP is associated with a significant learning curve regarding peri-interventional complications [7], TEE-guided TSP appears to be less dependent on experience [9]. It is likely that the improved visualization of the transseptal needle during TSP can be especially beneficial for less experienced interventionalists. However, as this study shows, even for experienced interventionalists, the additional use of TEE appears to lower the risk of pericardial tamponade and effusion. In this context, the enhanced visualization of patient-specific cardiac anatomy likely also plays a significant role. Even experienced interventionalists can more easily identify suboptimal or risky positions of the transseptal needle using TEE compared to fluoroscopy alone [4].

However, it remains unclear whether all patients benefit equally from the additional use of TEE during TSP. Given the extra personnel and structural resources required for TEE, it would be valuable to identify specific patient groups and electrophysiological procedures that gain the most in terms of procedural safety from this additional measure.

Furthermore, the potential risks associated with TEE should not go unmentioned. TEE is an invasive procedure, which can be associated with complications such as mucosal bleeding or, in the worst-case scenario, hollow organ perforations [14]. Interestingly, in this study, the risk associated with TEE was found to be minimal. The TEE-related complications were within the expected low range when compared to the existing literature [14]. A possible explanation for this is that only minimal probe movement in the esophagus is necessary to exclude intracardiac thrombus and, particularly, to guide TSP. As a result, performing TEE during electrophysiological procedures can be classified as a low-risk TEE intervention [14].

This study now shows that peri-interventional TEE, similar to what is already known for ICE in electrophysiological interventions [5], is associated with a clinically and statistically significant lower risk of pericardial effusion and tamponade.

To enhance patient safety during TSP in electrophysiological procedures, additional interventional echocardiographic imaging should be used alongside fluoroscopy. Whether this imaging is performed intracardially or transesophageally may depend on the specific clinical setting. While the use of ICE has the advantage over TEE of not necessarily requiring an additional interventionalist during the procedure, ICE is associated with higher costs [5,11], the need for an additional femoral venous access, and consequently, an increased risk of vascular access complications [5,15,16].

It remains unclear which of the two interventional echocardiography techniques should be preferred, as direct comparisons are currently lacking [17]. A direct comparison between TEE and ICE in the future would therefore be desirable.

### Limitations

The registry trial design carries the inherent limitations typical of this study type. Although patients were treated with or without additional TEE based on the timing of their presentation at our center, this process does not constitute randomization. Consequently, the study is non-confirmatory, and further prospective, randomized trials are needed to provide more definitive conclusions in this area. Another potential limitation of the study is the lack of recording the number of steam pops per procedure.

## 5. Conclusions

The use of additional TEE guidance during TSP appears to reduce the incidence of pericardial effusion and tamponade in electrophysiological procedures, thereby enhancing patient safety. As a result, TSP should be performed with additional echocardiographic guidance in electrophysiology. However, which patients benefit most from this approach needs to be investigated in further studies.

## Figures and Tables

**Table 1:** Overview of the baseline characteristics of both study groups.

	Total *n* = 932	TEE Group *n* = 443	Fluoroscopy Group *n* = 489	*p*-Value
Age, mean ± SD [years]	68.5 ± 11.3	68.1 ± 11.8	68.8 ± 11.0	0.384
Female, *n* (%)	367 (39.4)	180 (40.6)	187 (38.2)	0.613
Male, *n* (%)	565 (60.6)	263 (59.4)	302 (61.8)	
BMI, mean ± SD [kg/m^2^]	28.7 ± 6.0	28.9 ± 6.3	28.6 ± 5.7	0.678
PTT, mean ± SD [seconds]	33.8 ± 7.9	32.8 ± 7.3	34.6 ± 8.4	<0.001
INR, mean ± SD	1.19 ± 0.34	1.21 ± 0.36	1.18 ± 0.32	0.032
Platelet count, mean ± SD [Giga/L]	223.1 ± 60.6	224.4 ± 59.1	222.1 ± 62.1	0.341
Oral anticoagulation, *n* (%)	795 (85.3)	384 (86.7)	411 (84.0)	0.231
Vitamin-K antagonist, *n* (%)	29 (3.1)	12 (2.7)	17 (3.5)	
Apixaban, *n* (%)	322 (34.5)	150 (33.9)	172 (35.2)	
Dabigatran, *n* (%)	33 (3.5)	17 (3.8)	16 (3.3)	
Edoxaban, *n* (%)	141 (15.1)	80 (18.1)	61 (12.5)	
Rivaroxaban, *n* (%)	270 (29.0)	125 (28.2)	145 (29.7)	
Antiplatelet agents, *n* (%)	182 (19.5)	73 (16.5)	109 (22.3)	0.132
ASS, *n* (%)	51 (5.5)	18 (4.1)	33 (6.7)	
Clopidogrel, *n* (%)	116 (12.4)	50 (11.3)	66 (13.5)	
ASS + Clopidogrel, *n* (%)	5 (0.5)	3 (0.7)	2 (0.4)	
ASS + Prasugrel, *n* (%)	9 (1.0)	2 (0.5)	7 (1.4)	
ASS + Ticagrelor, *n* (%)	1 (0.1)	0 (0.0)	1 (0.2)	

ASS, acetylsalicylic acid; BMI, body mass index; INR, international normalized ratio; PTT, partial thromboplastin time; SD, standard deviation.

**Table 2:** Overview of the procedural data of both study groups.

	Total *n* = 932	TEE Group *n* = 443	Fluoroscopy Group *n* = 489	*p*-Value
Count of transeptal punctures ^1^				
Total (per group), *n*	1077	521	556	0.104
Mean (per patient) ± SD	1.16 ± 0.36	1.18 ± 0.38	1.14 ± 0.34	0.101
Maximum transseptal sheath diameter				
8.5 French, *n* (%)	477 (51.2)	222 (50.1)	255 (52.1)	0.289
12+ French, *n* (%)	455 (48.8)	221 (49.9)	234 (47.8)	
Treated tachycardia				
Atrioventricular reentry tachycardia, *n* (%)	6 (0.6)	3 (0.6)	3 (0.6)	
Left atrial flutter, *n* (%)	55 (5.9)	24 (5.4)	31 (6.3)	
Left focal atrial tachycardia, *n* (%)	32 (3.4)	15 (3.4)	17 (3.4)	0.022
Atrial fibrillation, *n* (%)	783 (84.0)	387 (87.4)	396 (80.9)	
Left ventricular tachycardia, *n* (%)	56 (6.0)	14 (3.1)	42 (8.6)	

^1^ The count of transseptal punctures refers to a single procedure.

**Table 3:** Logistic regression model with backward elimination to analyze risk factors associated with the occurrence of periprocedural pericardial tamponade.

Parameter	Odds Ratio	Confidence Interval	*p*-Value
TEE-guided puncture	0.103	0.013–0.819	0.032
Count of transseptal punctures [per puncture]	4.257	1.169–15.499	0.028

The following parameters were originally included in this regression model: TEE-guided puncture, age [per years], female sex [in comparison to male sex], body mass index [kg/m^2^], international normalized ratio [per one unit], partial thromboplastin time [per seconds], treated tachycardia, count of transseptal punctures [per puncture], transseptal sheath diameter 12+ French [in comparison to 8.5 French]. Omnibus test *p* = 0.004; Hosmer and Lemeshow test *p* = 0.542.

**Table 4:** Logistic regression model with backward elimination to analyze risk factors associated with the occurrence of periprocedural pericardial effusion.

Parameter	Odds Ratio	Confidence Interval	*p*-Value
TEE-guided puncture	0.466	0.246–0.883	0.019
Body mass index [kg/m^2^]	0.932	0.875–0.992	0.028
Count of transseptal punctures [per puncture]	2.681	1.376–5.226	0.004

The following parameters were originally included in this regression model: TEE-guided puncture, age [per years], female sex [in comparison to male sex], body mass index [kg/m^2^], international normalized ratio [per one unit], partial thromboplastin time [per seconds], treated tachycardia, count of transseptal punctures [per puncture], transseptal sheath diameter 12+ French [in comparison to 8.5 French]. Omnibus test *p* < 0.001; Hosmer and Lemeshow test *p* = 0.850.

**Table 5:** Logistic regression model with backward elimination to analyze risk factors for the need of periprocedural transfusion.

Parameter	Odds Ratio	Confidence Interval	*p*-Value
TEE-guided puncture	0.132	0.016–1.095	0.061
Age [per years]	1.092	0.998–1.196	0.056
Count of transseptal punctures [per puncture]	6.652	1.601–27.648	0.009

The following parameters were originally included in this regression model: TEE-guided puncture, age [per years], female sex [in comparison to male sex], body mass index [kg/m^2^], international normalized ratio [per one unit], partial thromboplastin time [per seconds], treated tachycardia, count of transseptal punctures [per puncture], transseptal sheath diameter 12+ French [in comparison to 8.5 French]. Omnibus test *p* = 0.002; Hosmer and Lemeshow test *p* = 0.948.

## Data Availability

The data presented in this study are available on request from the corresponding author. The data are not publicly available due to data privacy law.

## Supplementary Materials

The following supporting information can be downloaded at: https://www.mdpi.com/article/10.3390/diagnostics14222495/s1, Table S1: Logistic regression model analyzing the risk factors for periprocedural pericardial tamponade occurrence. Table S2: Logistic regression model analyzing the risk factors for periprocedural pericardial effusion occurrence. Table S3: Logistic regression model analyzing the risk factors for the need of periprocedural transfusion.
