# Supplementary material for: Transesophageal Echocardiography-Guided Transseptal Puncture Reduces Pericardial Tamponade in Electrophysiological Procedures
Source: Diagnostics (Basel). 2024 Nov 8;14(22):2495. doi: 10.3390/diagnostics14222495 (PMC11592922; doi:10.3390/diagnostics14222495)
Supplement: Supplementary file 1 [file diagnostics-14-02495-s001.zip › diagnostics-3279394-supplementary.pdf]

Supplements - Transesophageal echocardiography guided transseptal puncture reduces pericardial tamponade in electrophysiological procedures

Table S1: Logistic regression model analyzing the risk factors for periprocedural pericardial tamponade occurrence.

Parameter	Odds ratio	Confidence interval	p-value
**TEE guided puncture**	0.082	0.009 – 0.741	0.026
**Age [per years]**	1.038	0.963 – 1.118	0.329
**Female sex [in comparison to male sex]**	2.785	0.695 – 11.159	0.148
**Body mass index [kg/m^2]**	0.971	0.857 – 1.101	0.651
**International normalized ratio [per one unit]**	1.855	0.531 – 6.482	0.333
**Partial thromboplastin time [per seconds]**	1.085	1.028 – 0.974	0.318
**Treated tachycardia**	-[1]	-[1]	0.859
**Count of transseptal punctures [per puncture]**	8.448	0.937 – 76.126	0.057
**Transseptal sheath diameter 12+ French [in comparison to 8.5 French]**	1.960	0.219 – 17.552	0.548

Omnibus test p = 0.047; Hosmer and Lemeshow test p = 0.998; [1]This categorical variable with multiple values and lack of significance is not shown for the sake of clarity.

Table S2: Logistic regression model analyzing the risk factors for periprocedural pericardial effusion occurrence.

Parameter	Odds ratio	Confidence interval	p-value
**TEE guided puncture**	0.440	0.229 – 0.847	0.014
**Age [per years]**	0.999	0.971 – 1.028	0.964
**Female sex [in comparison to male sex]**	1.667	0.889 – 3.127	0.111
**Body mass index [kg/m^2]**	0.934	0.878 – 0.995	0.034
**International normalized ratio [per one unit]**	1.158	0.431 – 3.110	0.772
**Partial thromboplastin time [per seconds]**	0.984	0.939 – 1.033	0.521
**Treated tachycardia**	-[1]	-[1]	0.787
**Count of transseptal punctures [per puncture]**	3.041	1.256 – 7.362	0.014
**Transseptal sheath diameter 12+ French [in comparison to 8.5 French]**	1.140	0.494 – 2.629	0.758

Omnibus test p = 0.018; Hosmer and Lemeshow test p = 0.413; [1]This categorical variable with multiple values and lack of significance is not shown for the sake of clarity.

Table S3: Logistic regression model analyzing the risk factors for the need of periprocedural transfusion.

Parameter	Odds ratio	Confidence interval	p-value
**TEE guided puncture**	0.088	0.008 – 0.924	0.043
**Age [per years]**	1.086	0.986 – 1.195	0.094
**Female sex [in comparison to male sex]**	3.381	0.652 – 17.545	0.147
**Body mass index [kg/m²]**	1.013	0.885 – 1.161	0.847
**International normalized ratio [per one unit]**	1.937	0.548 – 6.855	0.305
**Partial thromboplastin time [per seconds]**	1.037	0.981 – 1.097	0.199
**Treated tachycardia**	-[1]	-[1]	0.789
**Count of transseptal punctures [per puncture]**	9.208	0.936 – 90.587	0.057
**Transseptal sheath diameter 12+ French [in comparison to 8.5 French]**	0.948	0.081 – 11.119	0.966

Omnibus test p = 0.021; Hosmer and Lemeshow test p = 0.987; [1]This categorical variable with multiple values and lack of significance is not shown for the sake of clarity.
